# The KCNH3 inhibitor ASP2905 shows potential in the treatment of attention deficit/hyperactivity disorder

**DOI:** 10.1371/journal.pone.0207750

**Published:** 2018-11-21

**Authors:** Shinji Takahashi, Makoto Ohmiya, Sokichi Honda, Keni Ni

**Affiliations:** Drug Discovery Research, Astellas Pharma Inc., Tsukuba, Ibaraki, Japan; Universidade do Estado do Rio de Janeiro, BRAZIL

## Abstract

N-(4-fluorophenyl)-N'-phenyl-N"-(pyrimidin-2-ylmethyl)-1,3,5-triazine-2,4,6-triamine [ASP2905] is a potent and selective inhibitor of the potassium voltage-gated channel subfamily H member 3 (KCNH3) that was originally identified in our laboratory. KCNH3 is concentrated in the forebrain, and its overexpression in mice leads to cognitive deficits. In contrast, *Kcnh3* knockout mice exhibit enhanced performance in cognitive tasks such as attention. These data suggest that KCNH3 plays important roles in cognition. Here we investigated the neurochemical and neurophysiological profiles of ASP2905 as well as its effects on cognitive function, focusing on attention. ASP2905 (0.0313 and 0.0625 mg/kg, po) improved the latent learning ability of mice, which reflects attention. Microdialysis assays in rats revealed that ASP2905 increased the efflux of dopamine and acetylcholine in the medial prefrontal cortex (0.03, 0.1 mg/kg, po; 0.1, 1 mg/kg, po, respectively). The activities of these neurotransmitters are closely associated with attention. We used a multiple-trial passive avoidance task to investigate the effects of ASP2905 on inattention and impulsivity in juvenile stroke-prone spontaneously hypertensive rats. ASP2905 (0.1 and 0.3 mg/kg, po) significantly prolonged cumulative latency as effectively as methylphenidate (0.1 and 0.3 mg/kg, sc), which is the gold standard for treating ADHD. Further, ASP2905, amphetamine, and methylphenidate significantly increased the alpha-band power of rats, suggesting that ASP2905 increases arousal, which is a pharmacologically important activity for treating ADHD. In contrast, atomoxetine and guanfacine did not significantly affect power. Together, these findings suggest that ASP2905, which acts through a novel mechanism, is as effective for treating ADHD as currently available drugs such as methylphenidate.

## Introduction

Attention deficit/hyperactivity disorder (ADHD), which is associated with a disorder of neuronal developmental, is characterized by major symptoms that first appear in children younger than seven years of age [1]. The symptoms are as follows: inattention (difficulty focusing and frequently losing objects), hyperactivity (lack of composure, difficulty remaining stationary), and impulsivity (executing uninhibited impetuous ideas, impatience).

The United States National Institutes of Mental Health has designated ADHD as one of the most common psychiatric disorders of children, and 6.4 million children were diagnosed with ADHD in 2011 [2]. Children with ADHD suffer impaired function at home and school as well as in their interactions with friends. Without treatment, ADHD exerts its effects from childhood to adulthood. The hyperactivity and impulsivity of children with ADHD are often relieved at approximately 11 to 13 years of age. However, approximately 70% of adolescent and 50% of adult patients with ADHD exhibit inattention [3, 4].

ADHD is treated combined pharmacotherapy and psychosocial therapy. Pharmacotherapy effectively controls symptoms and causes discrete changes in behavior, cognition, learning, and personal relationships. The psychostimulant methylphenidate, the drug of first choice, mitigates symptoms such as hyperactivity, attention deficit, impulsivity, and anxiety. Unfortunately, methylphenidate, which relieves 70% to 80% of symptoms in children with ADHD [5], may be easily abused and is therefore classified as a Schedule II controlled substance by the United States Drug Enforcement Agency and the Convention on Psychotropic Substances.

Potassium channels comprise transmembrane proteins that selectively pass potassium ions through the membrane and play important roles in regulating membrane potential. Through regulation of the frequency and duration of the action potential, potassium channels contribute to the mechanism of neurotransmission of central and peripheral nerves, pace making by cardiac cells, and muscle contraction. Voltage-dependent potassium channels open when the membrane potential is depolarized and discharge potassium ions from inside the cell to the extracellular milieu, which induces recovery (repolarization) of membrane potential. Therefore, opening potassium channels attenuates the excitability of neurons and muscle cells [6].

Potassium voltage-gated channel subfamily H member 3 (KCNH3) is expressed in certain areas of the brain that may help mediate learning and memory. This is reflected by the relatively high levels of KCNH3 in the hippocampus and cerebral cortex [7], which are strongly associated with learning and memory [8]. Moreover, transgenic mice overexpressing KCNH3 in these regions are learning-impaired, whereas *Kcnh3* knockout (KO) mice show improved performance in several behavioral tasks related to certain cognitive domains such as attention, working memory, and spatial memory [9]. Moreover, impaired long-term potentiation occurs in hippocampal slices from transgenic mice overexpressing KCNH3, whereas KO mice exhibit enhanced neuronal excitation when hippocampal slices are assessed in electrophysiological experiments [9]. In contrast, inhibition of KCNH3 in primary cultures of hippocampal neurons attenuates spontaneous inhibitory postsynaptic currents, suggesting that inhibition of KCNH3 inhibits GABAergic transmission [10]. We therefore reasoned that an inhibitor of KCNH3 might enhance attention and serve to treat inattention exemplified by ADHD.

The novel, potent and selective inhibitor of KCNH3 (4-fluorophenyl)-N'-phenyl-N"-(pyrimidin-2-ylmethyl)-1,3,5-triazine-2,4,6-triamine (ASP2905) ameliorates similar impairments in rodents subjected to certain cognitive tasks [10] and is therefore a strong candidate as a therapeutic. Here we used rats to study the effects of ASP2905 on neurotransmission in the medial prefrontal cortex and on the quantitative electroencephalogram (qEEG). Further, we tested the effect of ASP2905 on the attentional behavior of normal mice as well as on the inattention and impulsivity of juvenile stroke-prone spontaneously hypertensive rats (SHRSP), which serve as an animal model of ADHD.

Spontaneously hypertensive rats (SHRs), which serve as the best validated model of ADHD [11–13], exhibit inattention, slow habituation, and impulsivity [14, 15]. In patients with ADHD, these impairments are not caused by pharmacological or surgical intervention. Juvenile rats with SHR are not yet hypertensive, because this condition develops after 3 months of age [16]. Further, juvenile SHRSPs, an SHR substrain, serve as an animal model of a developmental disorder resembling ADHD [17]. For example, these rats exhibit abnormal brain function, such as decreased dopamine (DA) transmission, cholinergic dysfunction, and decreased local cerebral blood flow, which reflect the symptoms of patients with ADHD [17]. Therefore, SHRSPs are suitable for screening compounds for treating ADHD.

## Material and methods

### Drugs and animals

ASP2905 hydrochloride or ASP2905 hemifumarate (Astellas Pharma Inc. Ibaraki, Japan) was suspended in 0.5% methylcellulose. Atomoxetine hydrochloride (Sequoia Research Product, Pangbourne, UK) was dissolved in distilled water, and amphetamine sulfate, guanfacine hydrochloride (Astellas Pharma Inc.), and methylphenidate hydrochloride (Sigma-Aldrich St. Louis, MO, USA) were dissolved in saline. We administered ASP2905 orally to ddY (Japan SLC, Shizuoka, Japan) mice (10 mL/kg), to juvenile stroke-prone spontaneous hypertensive rats (SHRSP) and Wistar-Kyoto (WKY; Hoshino Laboratory Animals, Ibaraki, Japan) rats (2 mL/kg), and to Wistar/ST and Sprague-Dawley (SD) (Japan SLC, Shizuoka, Japan) rats (1 mL/kg). Methylphenidate was administered subcutaneously to juvenile SHRSP and WKY rats (2 mL/kg) and to SD rats (1 mL/kg). Atomoxetine was administered orally, and amphetamine and guanfacine were injected subcutaneously into SD rats (1 mL/kg). The doses of ASP2905, atomoxetine, guanfacine, and methylphenidate are expressed as the respective free base. All animal experiments were prospectively approved by the Institutional Animal Care and Use Committee of Astellas Pharma Inc., which is accredited by the Association of Assessment and Accreditation of Laboratory Animal Care (AAALAC).

### Water finding task (WFT)

Male ddY mice (Japan SLC) aged 5 weeks, beginning with 28 mice per group, were subjected to the WFT. After selecting mice according to the exclusion criteria described below, the final number per group ranged from 21 to 23. The apparatus comprised an open field (30 × 50 cm, 15 cm high) and an alcove (10 × 10 cm, 10 cm high) in the middle of one of the enclosure’s long walls. A drinking tube was inserted into the center of the alcove ceiling with its tip 5.5 cm (training trial) or 8 cm (test trial) above the floor. The experiments were performed according to a published method [18].

When the training trial commenced (day 1), ASP2905 was administered orally (0.0078, 0.0156, 0.0313 and 0.0625 mg/kg), and mice were then returned to their home cage. After 30 min, the mice were placed in the open field where they freely explored the training apparatus for 3 min. Before the training trial, mice were not deprived of water, and were therefore not motivated to drink water. A water tube was placed in the alcove; however, water was not delivered from the drinking tube. During the training trial, no significant difference was observed between control and ASP2905-treated groups in ambulation (line crossing, data not shown). The mice were returned to their home cage immediately after training, where they were deprived of water until the test trial.

We excluded mice from the test trial if they failed to explore after 3 min or approached the drinking tube fewer than two times. The mice were again placed individually in the test apparatus during the test trial (day 2). Starting latency (SL), entering latency (EL), and drinking latency (DL) were defined as the times until a mouse moved out of the corner, entered the alcove, and drank the water, respectively. The cutoff time was 600 s. Finding latency (FL = DL–EL) represents the index of latent learning.

### Five-trial passive avoidance task (5T-PAT)

Male SHRSP and WKY rats aged 4 weeks were subjected to the 5T-PAT using a step-through passive avoidance apparatus (control units, PA-2030A; light-dark box, PA3001A; O’Hara Ika-Sangyou Co. Ltd., Japan). The experiment described below was performed using a slightly modified published method [19]. Rats were administered ASP2905 (0.03, 0.1, 0.3 mg/kg) or methylphenidate (0.03, 0.1, 0.3 mg/kg) and then returned to their home cage. Thirty minutes later, they were gently placed in the illuminated room. When they entered the dark room, an electric current (intensity 0.15 mA; delay, 3 s; duration 1 s) was delivered through the metal grid on the floor. The rats were then removed from the apparatus and returned to their home cage. The time until the rat entered the dark compartment was defined as the step-through latency, and measurements were conducted for ≤180 s. The trial was terminated if latency exceeded 180 s, and latency in the following trials was recorded as 180 s. The acquisition trial (the procedure after administration of drugs) was repeated 5 times at approximately 1-min intervals, and the cumulative latency between the second and fifth trial was determined. Ten rats were included in each group.

### Microdialysis

Microdialysis experiments were performed according to a published method [20]. Male Wistar/ST rats aged 9–10 weeks were anesthetized with sodium pentobarbital. A stereotactic apparatus was used to implant a microdialysis guide cannula into the medial prefrontal cortex (mPFC) (anteroposterior, +3.0 mm; mediolateral, –0.8 mm; dorsoventral, –0.5 mm for DA and –1.0 mm for acetylcholine [ACh]). Rats were allowed to recover from surgery for at least 4 days. On the day of the microdialysis measurements, each rat was placed into a plastic cage, and a dialysis probe (CMA8309564, 4 mm for DA, CMA8309563, 3 mm for ACh; CMA microdialysis, Kista, Sweden) was inserted into the guide cannula. The dialysis probe was perfused (1.0 μL/min and 2.0 μL/min for DA and ACh, respectively) with modified Ringer’s solution (NaCl, 140 mM; KCl, 4.0 mM; CaCl_2_·2H_2_O, 2.4 mM; and MgCl_2_·6 H_2_O, 1.0 mM) containing physostigmine (10 μM) for ACh. Dialysates were collected every 30 min and 20 min for DA and ACh, respectively, starting 3–4.5 h after probe insertion. Three samples were collected, and the values were averaged and defined as baseline. ASP2905 was then orally administered to rats (0.01, 0.03 and 0.1 mg/kg [DA] or 0.1 and 1 mg/kg (ACh). Dialysates were collected for 180 min and 100 min for DA and ACh, respectively.

Extracellular DA levels in the mPFC were measured using an Eicom HTEC-500 (Eicom, Kyoto, Japan) high-pressure liquid chromatography (HPLC) system equipped with an electrochemical detector (ECD). DA was separated using a reverse-phase Eicompak PP-ODS column that was eluted with 1% methanol, 500 mg/mL sodium 1-decanesulfonate, and 50 mg/mL EDTA (pH 6.0) at 500 μL/min at 25°C. The concentration of DA was measured by setting the working electrode of the ECD to +400 mV vs a silver/silver-chloride reference electrode. The HPLC-ECD unit was calibrated using a standard DA solution before each experiment.

Extracellular ACh levels in the mPFC were quantified using a combination of HPLC, an enzymatic assay, and an ECD. A solution of 100 mM KHCO_3_ containing 300 mg/L sodium 1-decanesulfonate and 50 mg/L EDTA delivered at 500 μl/min. Isopropylhomocholine (IPHC) (40 μL, 100 nM) served as an internal standard. Samples (40 μL) were collected, and 65 μL was injected into the HPLC. After separation using a column packed with a polystyrene resin (AC-GEL; Eicom), ACh was converted to H_2_O_2_ using a postcolumn enzyme reactor (AC-ENZYMPAK; Eicom) containing immobilized acetylcholinesterase and choline oxidase. The separation column and postcolumn reactor were maintained at 33°C. H_2_O_2_ was detected using an ECD-300 (Eicom) set to +450 mV vs a silver/silver-chloride reference electrode. The HPLC-ECD unit was calibrated before each experiment using ACh and IPHC standards. Ten animals were included in each group.

### Electroencephalogram (EEG) analysis

The experiments were performed according to a published method with some modifications to optimize the analysis [21]. Male SD rats aged 8 weeks were anesthetized using isoflurane and then placed into the stereotactic apparatus. After inducing local anesthesia with lidocaine, an incision was made to expose the skull. The bregma was identified, and stainless-steel screw electrodes with wire leads were implanted epidurally over the vertex (AP, –1.0; ML, −1.0; and DV, −1.0), cerebellum (ground), and frontal sinus (reference). Lead wires were connected to the pedestal, and dental cement was used to secure the entire assembly to the skull. Recording was conducted for at least 10 days postsurgery (10–24 weeks of age).

After acclimation, rats were placed in a recording chamber in an electrically shielded field. The signal from the electrode first passed through the miniature preamplifier attached to the rat’s head (JH-110J; Nihon Kohden Corporation, Tokyo, Japan) and then to a signal selector (JB-120J; Nihon Kohden Corporation). The signal value was stored using a data acquisition system (Micro1401 hardware and Spike2 software; Cambridge Electric Design, Ltd., Cambridge, UK). If animals were used repeatedly, EEGs were administered at least after one-week intervals.

The sequencer file comprised a series of 200-paired white-noise stimuli presented 500 ms apart at 75 dB (10 ms in duration) with 9- to 13-s randomized interstimulus intervals. After recording sessions of a 40 min acclimation phase, each drug was administered to the rats. EEG recording was performed 60 min after oral administration of ASP2905 (1, 3, 10 mg/kg) and atomoxetine (3, 10, 30 mg/kg), or 30 minutes after subcutaneous administration of amphetamine (0.3, 1, 3 mg/kg), methylphenidate (0.3, 1, 3 mg/kg), or guanfacine (0.03, 0.1, 0.3 mg/kg). Raw EEG data were filtered between 1 Hz and 1,000 Hz. Individual sweeps were rejected for movement artifacts according to the criterion of 2 × root-mean squared amplitude per rat.

Time-frequency decomposition of the EEG signal was determined using the EEGLAB toolbox in MATLAB (The MathWorks, Inc., Natick, MA, USA). Signal trial epochs between –1.0 s and 2.0 s relative to the sound trigger were extracted from the continuous data sampled at 1,000 Hz. Fast Fourier transform was used to generate power spectra from the EEG signals for consecutive 128-ms windows. The electrical power spectra were divided into predefined frequency ranges (delta, 1–4 Hz; theta, 4–7 Hz; alpha, 7–13 Hz; and beta1, 13–30 Hz). The area under the curve (AUC) for each alpha power value was calculated from the power-frequency plot. The difference between post- and pretreatment AUC values indicates the effect of each drug. Each group included 5 rats.

### Statistical analysis

Statistical analysis was performed using SAS software (SAS 8.2, SAS Institute Japan, Tokyo, Japan). Data are expressed as the mean ± standard error of the mean (SEM). For WFT, microdialysis, and EEG data, statistical significance was evaluated using 1-way ANOVA followed by Dunnett’s multiple comparison test, and *p* < 0.05 indicates a statistically significant difference. The *p* values of the 5T-PAT were calculated using the Student *t* test (comparisons between WKY and SHRSP groups) and 1-way ANOVA followed by Dunnett's multiple comparison test (effect of drugs).

## Results

### ASP2905 decreases the finding latency of mice in the WFT

**Fig 1:**
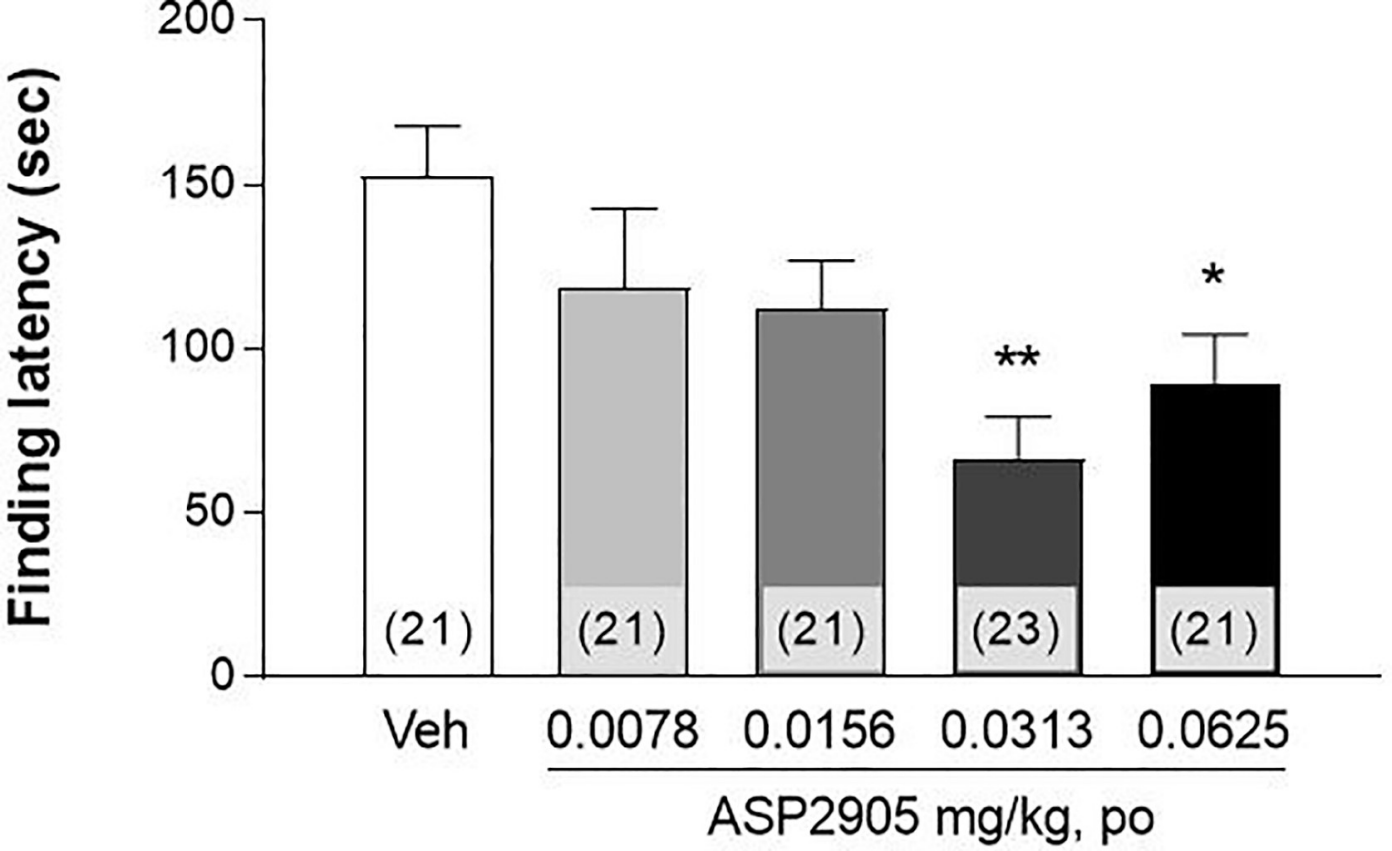
Effect of ASP2905 on the finding latency of mice in the water finding task. Data are expressed as the mean ± SEM of finding latency. The numbers in parentheses indicate the number of mice in each group. *p<0.05, **p<0.01 vs vehicle group (Dunnett’s test).

Normal mice found the water nozzle with a finding latency (FL) of 153 ± 15 s. FL was significantly shorter when mice were treated with ASP2905 (F [4, 102] = 3.866, *p* = 0.0058; one-way ANOVA; 0.0313 and 0.0625 mg/kg, *p* = 0.0013 and 0.0312, respectively; Dunnett's multiple comparison test) (Fig 1).

### ASP2905 and methylphenidate prolong the cumulative latencies of SHRSP rats subjected to the 5T-PAT

**Fig 2:**
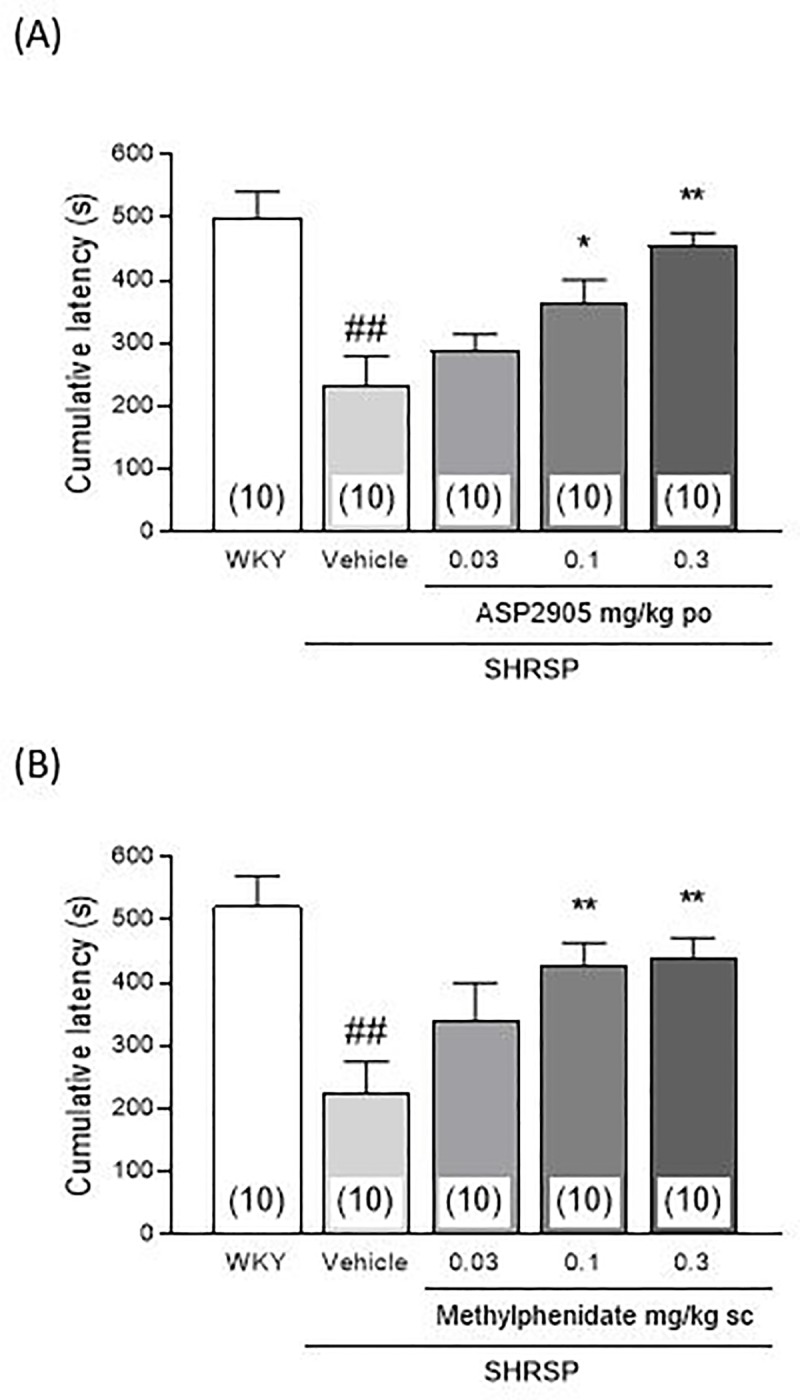
**Effect of ASP2905 (A) and methylphenidate (B) on cumulative latency in the 5-trial passive avoidance task administered to juvenile SHRSP.** Cumulative latency data are expressed as the mean ± SEM. The numbers in parentheses indicate the number of rats in each group. *p <0.05, **p <0.01 vs the vehicle-treated SHRSP group (Dunnett’s test); ^*##*^*p* <0.01 vs the WKY group (Student *t* test).

The cumulative latencies of juvenile SHRSP were significantly shorter compared with those of WKY rats (Fig 2). ASP2905 (0.1 and 0.3 mg/kg) significantly increased the cumulative latencies of juvenile SHRSP compared with controls (Fig 2A, F [3, 36] = 7.783, *p* = 0.0004, 1-way ANOVA; 0.1 and 0.3 mg/kg, *p* = 0.0303 and 0.0002, respectively, Dunnett's multiple comparison test). Methylphenidate (0.1 and 0.3 mg/kg) significantly increased the cumulative latencies of juvenile SHRSP compared with controls (Fig 2B, F [3, 36] = 4.766, *p* = 0.0067, 1-way ANOVA; 0.1 and 0.3 mg/kg, *p* = 0.0097 and 0.0051, respectively, Dunnett's multiple comparison test).

### ASP2905 administered to rats increases extracellular levels of DA and ACh in the mPFC

**Fig 3:**
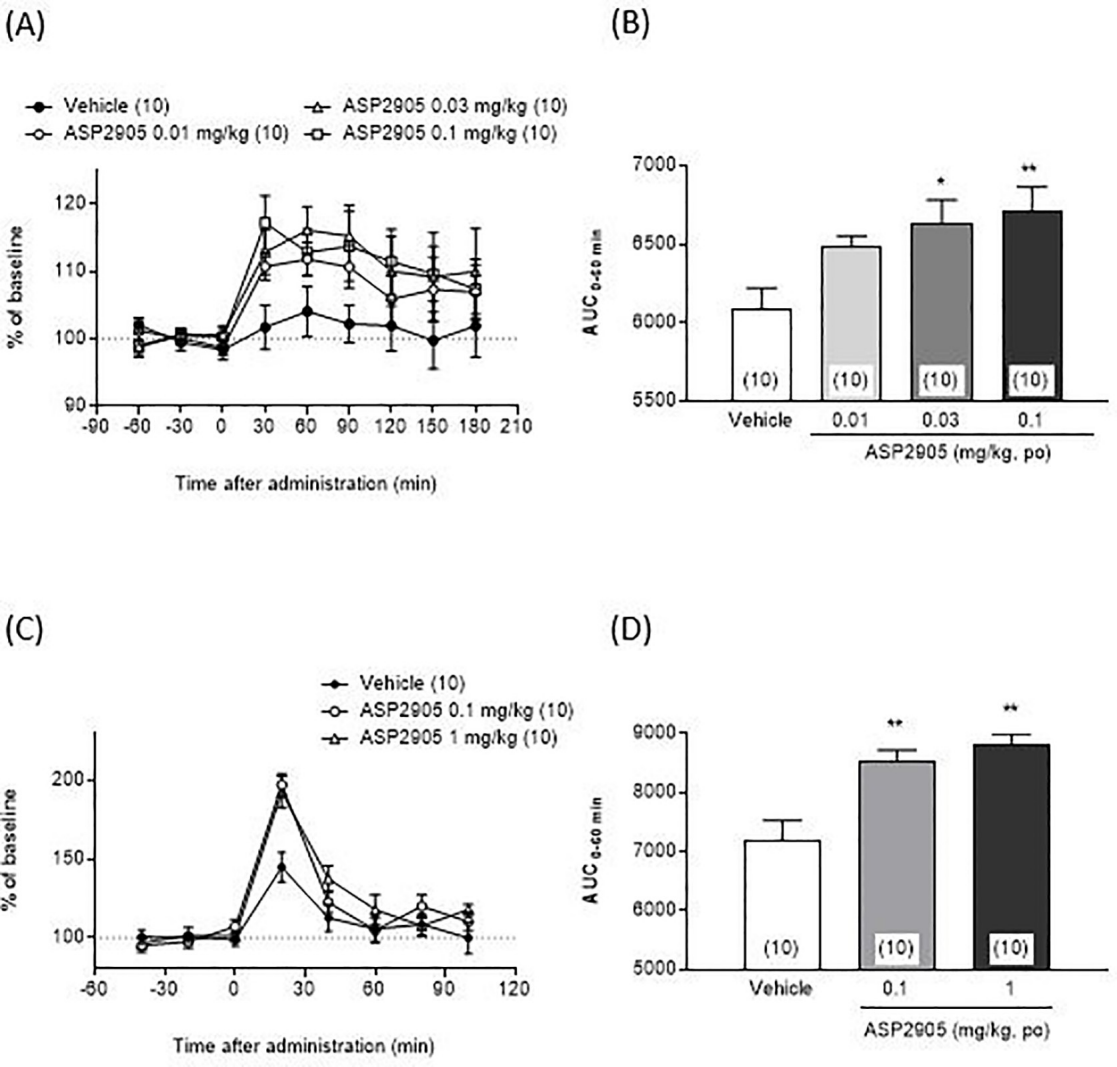
**Effects of ASP2905 on the extracellular levels of DA (A, B) and ACh (C, D) in the rat mPFC.** Data are expressed as the mean ± SEM of percentages of the baseline (A and C) and AUCs 60 min after administration of ASP2905 (B and D). The numbers in parentheses indicate the number of rats in each group. For DA, animals with basal levels differing by ± 10% from the mean were excluded from the analysis. The IPHC values for ACh were compensated by the IPHC value of the third point acquired before administering ASP2905, and the value of ACh was corrected using the compensated value of IPHC. When the basal level differed by >30% from the mean, the data for that animal were excluded from the analysis. *p<0.05, **p<0.01 vs vehicle-treated group (Dunnett’s test). A) Extracellular levels of DA after administration of ASP2905. B) AUCs of the levels of DA in the mPFC 60 min after administration of ASP2905. C) Extracellular ACh levels after administration of ASP2905. D) AUCs of the levels of Ach in the mPFC 60 min after administration of ASP2905.

Oral administration of ASP2905 increased the extracellular levels of DA (Fig 3A and 3B). The AUCs 60 min after administration of ASP2905 (0.03 and 0.1 mg/kg) were significantly greater compared with those after administration of saline (F [3,36] = 4.662, *p* = 0.0075, 1-way ANOVA; 0.03 and 0.1 mg/kg, *p* = 0.0131 and 0.0043, respectively, Dunnett's multiple comparison test). Further, oral administration of ASP2905 increased the levels of extracellular ACh (Fig 3C and 3D). The AUCs 60 min after administration of ASP2905 (0.1 and 1.0 mg/kg) were significantly greater compared with those after the administration of saline (F [2, 27] = 12.6, *p* = 0.0001, 1-way ANOVA; 0.1 and 1.0 mg/kg, *p* = 0.0014 and 0.0001, respectively, Dunnett's multiple comparison test).

### ASP2905 administered to rats increases alpha power

**Fig 4:**
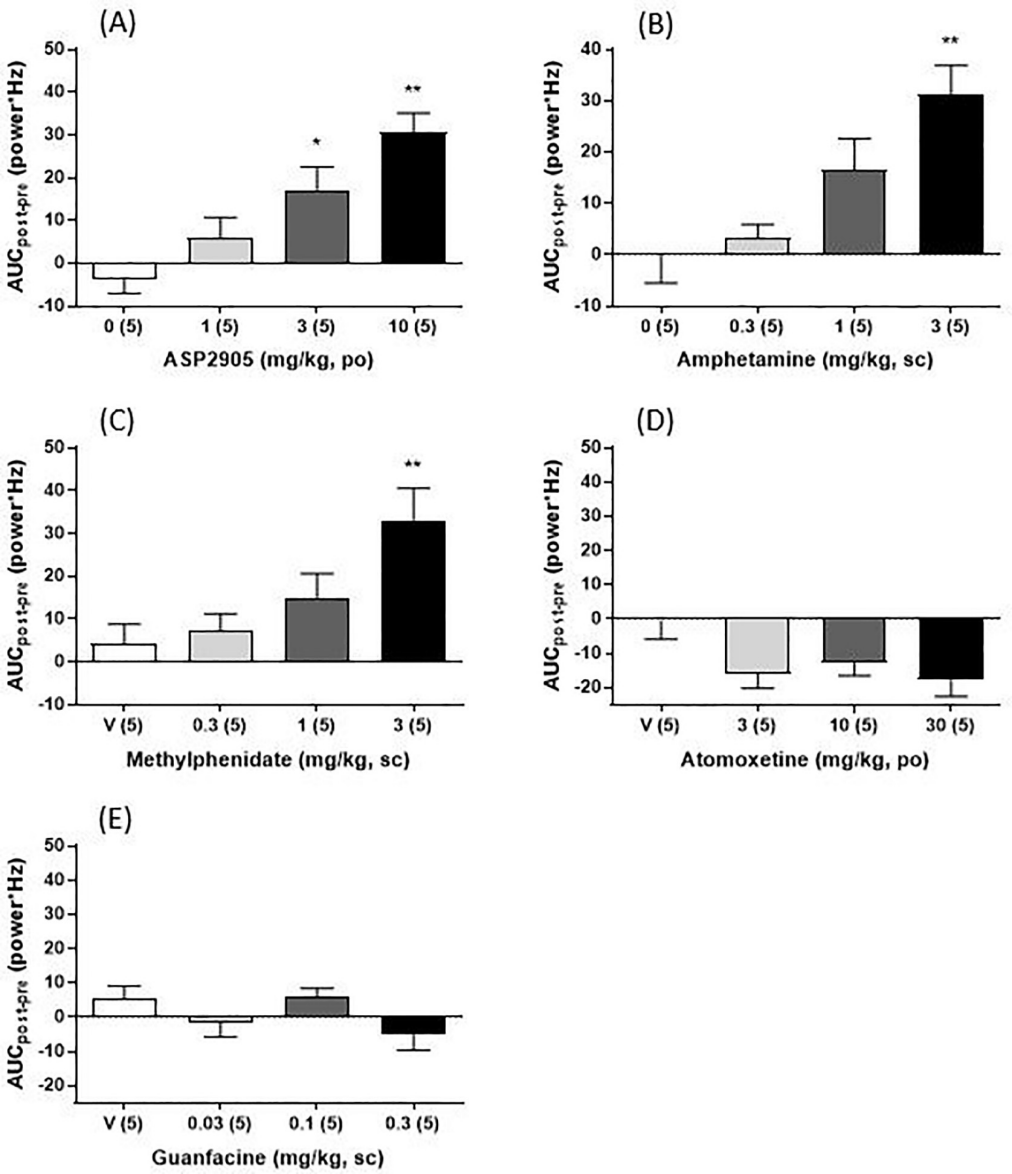
**Effect of ASP2905 (A), amphetamine (B), methylphenidate (C), atomoxetine (D), and guanfacine (E) on alpha power in rats.** Alpha power is quantified as an AUC of the difference between powers before and after administering a drug. Data are expressed as the mean ± SEM. The numbers in parentheses indicate the number of rats in each group. *p<0.05, **p<0.01 vs vehicle-treated group (Dunnett’s test).

**Fig 5:**
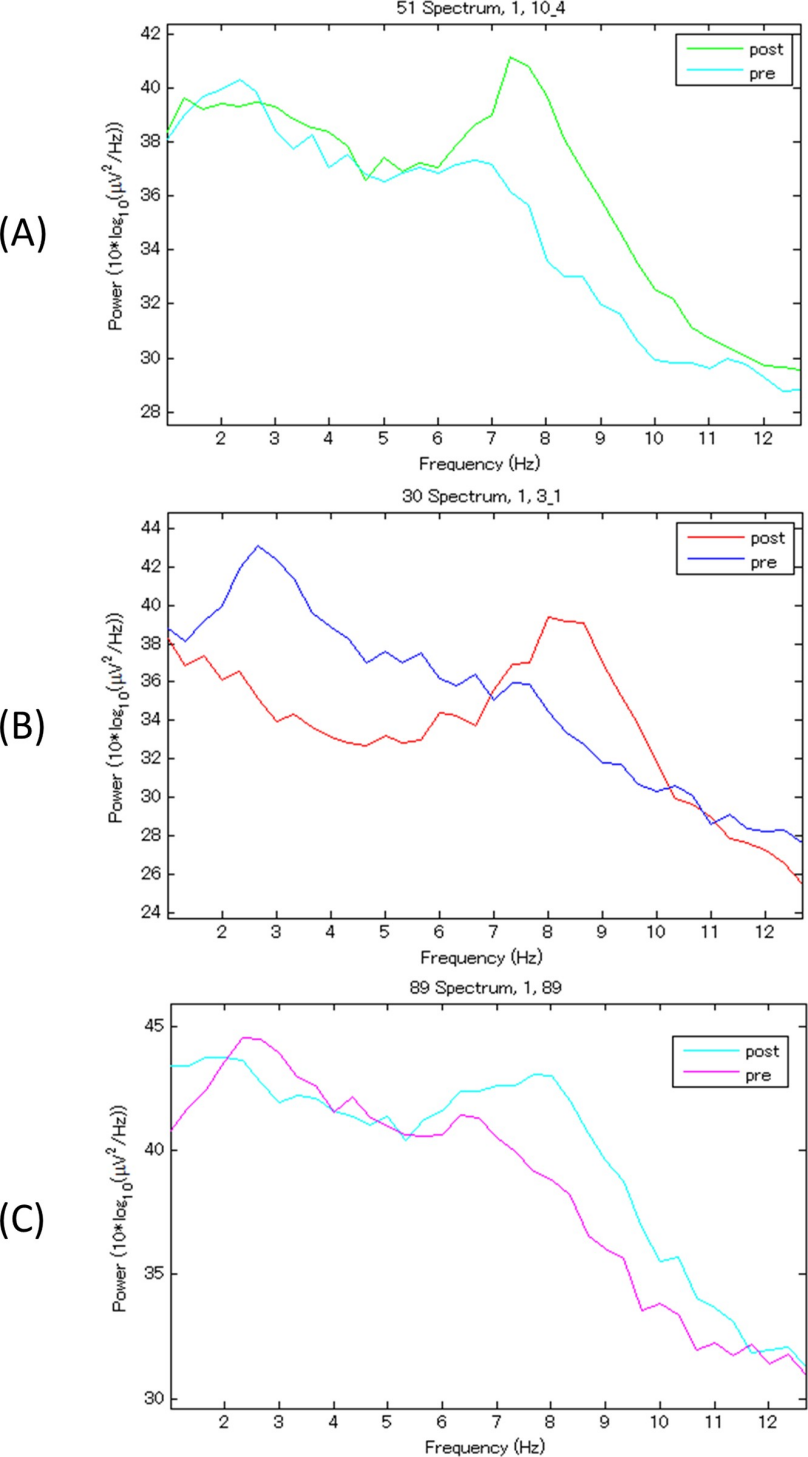
EEG spectra acquired before and after treatment of rats with ASP2905, amphetamine, or methylphenidate. Representative EEG spectra. Each graph indicates EEG power spectra from 1 Hz to 13 Hz of each rat before and after treatment with A) ASP2905 (10 mg/kg po), B) amphetamine (3 mg/kg sc), and C) methylphenidate (3 mg/kg sc).

EEG analysis revealed significantly increased alpha power in rats administered ASP2905, amphetamine, or methylphenidate (Fig 4A–4C). ASP2905 (F [3, 16] = 10.53, *p* = 0.0005, 1-way ANOVA; 3, 10 mg/kg, *p* = 0.0142, 0.0002, respectively, Dunnett’s multiple comparison test), amphetamine (F [3, 16] = 7.773, *p* = 0.0020, 1-way ANOVA;3 mg/kg, *p* = 0.0014, Dunnett’s multiple comparison test) and methylphenidate (F [3, 16] = 5.409, *p* = 0.009, 1-way ANOVA;3 mg/kg, *p* = 0.0057, Dunnett’s multiple comparison test) increased alpha power. Further, amphetamine tended to decreased theta power (Fig 5). In contrast, atomoxetine (F [3, 16] = 2.968, *p* = 0.0633, 1-way ANOVA) (Fig 4D) and guanfacine (F [3, 16] = 2.098, *p* = 0.1407, 1-way ANOVA) (Fig 4E) did not significantly increase alpha power.

## Discussion

The major findings of the present study are as follows: (1) ASP2905 enhanced attention in normal mice and ameliorated inattention and impulsivity in juvenile SHRSP, which serve as a model of ADHD; (2) ASP2905 increased the extracellular levels of DA and ACh in the rat mPFC; and (3) ASP2905 increased EEG alpha power in rats. Together, these findings demonstrate that ASP2905 has potential as a new medication to treat patients with ADHD.

Here we evaluated latent learning, which is related to attention, using the WFT [22, 23]. In this task, mice are not motivated to learn the location of a water source, because they are not water-deprived before the training trial. Mice are deprived of water immediately after the training trial to promote the recall of the location of the water delivery tube in the test trial. Thus, the FL in the WFT provides a measure of latent learning, spatial attention, or both.

For example, nicotine, which enhances attention, shortens the FL of mice in the WFT [24]. Further, a deficit in noradrenergic nerve function, which modulates arousal levels, lengthens the FL of mice [23]. Our present findings suggest that ASP2905 increased the levels of attention and arousal, because ASP2905 shortened FL in the WFT. Therefore, these findings further support the conclusion that ASP2905 may serve as an effective agent for treating patients with ADHD.

We used normal animals to evaluate the effects of ASP2905 on performance in the WFT to circumvent the potentially confounding effects of the pathophysiological phenotypes represented by animal models of disease. ASP2905 enhanced the cognitive function of normal animals, suggesting its utility for treating patients with diverse cognitive dysfunctions through enhancement of the normal functions of the remaining active neurons.

SHRs are frequently used as an animal model of ADHD [11–13]. Fox et al. found that SHRs are inattentive, impulsive, or both, as revealed by their performance in the 5T-PAT [19]. Rats usually chose a dark compartment when given a choice between entering an illuminated or dark compartment. When an electrical shock is delivered from a floor grid in the passive avoidance task, rats will learn to avoid entering a dark compartment. Here, when we used a stimulus with very mild amplitude and duration, rats had difficulty in learning, unless they were repeatedly administered an adverse stressor.

SHR is one of strain which shows inattention and impulsivity. In particular, juvenile SHRs exhibit increases in impulsive and their cumulative latencies are decreased compared with Wister-Kyoto or Wistar rats [19]. Therefore, this model can effectively evaluate the inattention and impulsivity of SHRs [19]. Moreover, the neuronal nicotinic receptor agonist ABT-418 and methylphenidate, both can improve clinical symptoms of ADHD, are effective in this model, therefore, this model is thought to be useful to evaluate novel compounds for treating ADHD.

Further, juvenile SHRSP, an SHR substrain, exhibits abnormal brain functions such as decreased DA transmission, cholinergic dysfunction, and decreased local cerebral blood flow, which reflect the symptoms of patients with ADHD [17]. We therefore reasoned that subjecting SHRSP to the 5T-PAT would represent a valid model for evaluating the efficacy of compounds for treating ADHD. Thus, the cumulative latencies of juvenile SHRSP in the present study were shorter compared with those of WKY rats, which serve as a model for inattention and impulsivity. Further, ASP2905 prolonged cumulative latency.

Performance in the 5T-PAT addresses normally slow learning and a memory response that is generally apparent in rat pups aged 2 to 4 weeks compared with older rats [25]. However, learning performance in the 5T-PAT of SHRSP subjected to a short, very mild adverse stimulus differed markedly from that of normal rats, due to the amplification of slow learning and memory response using juvenile SHRSP. Thus, fewer trials are required using the 5T-PAT because of the rats’ increased sensitivities, allowing the use of a relatively short (1 min) intertrial interval as previously described [19]. These conditions appear optimal for examining short-term memory effects and evaluating other components of the repeated acquisition task, such as response inhibition (i.e. repeated avoidance of the dark compartment).

In the 5T-PAT, juvenile SHRSP exhibited shorter cumulative latencies than WKY rats. Because impulsivity, impaired attention, and learning are common clinical features of patients with ADHD [26], the 5T-PAT simulates certain behavioral components of this disorder. Here, ASP2905 prolonged the cumulative latency of juvenile SHRSP in this task, suggesting that ASP2905 accelerated the avoidance response. Given that delayed acquisition of the avoidance response reflects inattention, impulsivity, or both in juvenile SHRSP, ASP2905 might be useful for managing these symptoms exhibited by patients with ADHD.

The psychostimulants methylphenidate and amphetamine are the drugs of choice for treating ADHD, because they mitigate the three core symptoms exhibited by 70%–80% of patients of all ages [5, 27]. We show here that methylphenidate prolonged cumulative latency in juvenile SHRSP, suggesting that this molecule might accelerate their avoidance response and that this model might serve to predict drugs that will be effective for treating ADHD. Further, in the present study, methylphenidate was effective at subcutaneous doses of 0.1 and 0.3 mg/kg. The clinically optimal effective oral dose range of methylphenidate for treating children with developmental disabilities and ADHD is 0.1 mg/kg to 1 mg/kg [28, 29]. For treating adults, an initial twice-daily oral dose of 5–10 mg is increased to 0.5–1 mg/kg for ADHD [30]. These doses are quite similar to those that were effective in the animal model employed here, supporting the conclusion that such a model is appropriate for preclinical assessment.

We found that ASP2905 induced a dose-dependent increase in the extracellular levels of DA and ACh in the mPFC. These doses are similar to the effective doses in the WFT and 5T-PAT. Because KCNH3 is expressed exclusively in forebrain areas such as the mPFC, modulation of KCNH3 activity might directly or indirectly affect the excitability of neurons restricted to the forebrain, including DA and ACh neurons that may modulate cognitive functions including attention. Therefore, we suggest that ASP2905 increases neurotransmission in the brain for the following reasons. First, we reported an association between KCNH3 activity and neural excitation [9]. Second, inhibition of KCNH3 activity decreases spontaneous inhibitory postsynaptic currents, suggesting that inhibition of KCNH3 decreases in GABAergic transmission [10].

DA regulates aspects of working memory, planning, and attention. For example, moderate enhancement of dopaminergic transmission in the mPFC improves learning and memory [31–33]. In particular, evidence indicates that the D1-type DA receptor expressed in the PFC mediates working memory [34]. Further, infusing the mPFC with a D1-receptor agonist enhances the accuracy of attentional performance of rats [35]. Moreover, drug-induced improvements in working memory are accompanied by a simultaneous increase in ACh efflux in the PFC [36] and hippocampus [37]. For example, ACh levels increase in the mPFC during the performance of the 5-choice serial reaction time task [38], and the tonic and phasic changes in ACh release are related to attention [39].

Local application of ACh enhances the attentional modulation of neuronal activity in the primary visual cortex, and the muscarinic antagonist scopolamine reduces the effects of attention in primates [40]. In humans, physostigmine, which affects the activities of the nicotinic and muscarinic acetylcholine receptors, improves selective attention during emotional processing and augments activity in the dorsolateral and medial prefrontal cortices, the orbitofrontal cortex, and anterior cingulate as well as temporal pole activity [41]. Further, physostigmine increases the effect of attention in the extrastriate occipital and prefrontal cortices and decreases activity in the superior-medial parietal cortex during performance of a working memory task [42]. These results suggest that ACh mediates attention among rodents, nonhuman primates, and humans. We therefore conclude that increased cholinergic and dopaminergic transmission enhances cognition, such as attention, which supports our findings showing the enhancing effects of ASP2905 on attention.

We also found that ASP2905, amphetamine, and methylphenidate increased the alpha power of rats. Amphetamine and methylphenidate increase qEEG alpha power, which is related to arousal in humans [43, 44] and mitigate the symptoms of ADHD, suggesting that ASP2905 will be useful for treating ADHD. The effect of ASP2905 on the qEEG of rats was similar to that of methylphenidate, which specifically increased alpha power. In contrast, amphetamine decreased theta power, suggesting that the effect of ASP2905 on qEEG more closely resembles that of methylphenidate. In contrast, atomoxetine and guanfacine did not increase the alpha power of rats. The efficacy of drugs used to treat ADHD depends, in part, on their abilities to increase arousal. However, atomoxetine is only weakly effective in this respect, and possibly weaker than stimulants that significantly increased alpha power in the present study.

The sedative α_2_-adrenoceptor agonist guanfacine [45] induces somnolence as one of its adverse effects [46]. These findings are consistent with our present results.

In EEG experiments, the effective doses of ASP2905 were higher than those in our other experiments. Compared with methylphenidate doses used in our behavioral studies, higher doses were required in experiments using rats, suggesting that our EEG experiments required relatively higher doses to detect significant responses. If so, this represents a limitation of our study. Another limitation is our use of only male mice and rats. One reason for this is that the estrous cycle can influence behavioral response in rats [47, 48]. Therefore, we used only males to exclude the potentially confounding influence of the estrous cycle.

## Conclusions

In conclusion, ASP2905 enhanced the attention of normal mice and mitigated the attention deficits and impulsivity of SHRSP. Further, in the mPFC, ASP2905 increased extracellular levels of the neurotransmitters DA and ACh, which modulate cognitive functions such as attention and working memory. ASP2905 increased alpha power in rats, which is related to arousal, and exerted effects on the qEEG response that were similar to those of methylphenidate, the drug of choice for treating patients with ADHD.

ASP2905 is a potent and selective inhibitor of KCNH3. Together, these findings show that ASP2905, which acts through a novel mechanism, is as effective as currently available drugs such as methylphenidate for treating patients with ADHD, and may provide physicians with a new treatment choice for these patients.
